# Risk of adverse pregnancy outcomes in women with periodontal disease and the effectiveness of interventions in decreasing this risk: protocol for systematic overview of systematic reviews

**DOI:** 10.1186/s13643-016-0195-7

**Published:** 2016-02-01

**Authors:** Sizzle F. Vanterpool, Kathleen Tomsin, Leticia Reyes, Luc J. Zimmermann, Boris W. Kramer, Jasper V. Been

**Affiliations:** Department of Pediatrics, Maastricht University Medical Center, P. Debyelaan 25, 6229 HX Maastricht, The Netherlands; Department of Neuroscience, School for Mental Health and Neuroscience (MHeNS), Maastricht University, Universiteitssingel 50, 6229 ER Maastricht, The Netherlands; Department of Pathobiological Sciences, School of Veterinary Medicine, University of Wisconsin–Madison, 2015 Linden Drive, Madison, WI 53706 USA; School for Oncology and Developmental Biology (GROW), Maastricht University, Universiteitssingel 40, 6229 ER Maastricht, The Netherlands; School for Public Health and Primary Care (CAPHRI), Maastricht University, Universiteitssingel 40, 6229 ER Maastricht, The Netherlands; Division of Neonatology, Sophia Children’s Hospital, Erasmus University Medical Center, Wytemaweg 80, 3015CN Rotterdam, The Netherlands

**Keywords:** Periodontal disease, Periodontitis, Pregnancy, Adverse pregnancy outcome, Preterm birth, Perinatal mortality, Maternal mortality, Treatment, Systematic review, Overview

## Abstract

**Background:**

Periodontal disease is an inflammatory disease of the tissues supporting the teeth. Women who have periodontal disease while pregnant may be at risk of adverse pregnancy outcomes. Although the association between periodontal disease and adverse pregnancy outcomes has been addressed in a considerable number of systematic reviews and meta-analyses, there are important differences in the conclusions of these reviews. Systematic reviews assessing the effectivity of various therapeutic interventions to treat periodontal disease during pregnancy to try and reduce adverse pregnancy outcomes have also arrived at different conclusions. We aim to provide a systematic overview of systematic reviews comparing the frequency of adverse pregnancy outcomes between women with and without periodontal disease and/or evaluating the effect of preventive and therapeutic interventions for periodontal disease before or during pregnancy on adverse pregnancy outcomes.

**Methods:**

We will include systematic reviews reporting on studies comparing adverse pregnancy outcomes: (i) between women with or without periodontal disease before (<6 months) or during pregnancy and/or (ii) according to preventive or therapeutic interventions for periodontal disease. Eligible interventions include (combinations of) the following: oral hygiene education, use of antibiotics, subgingival scaling, and root planing. For preventive and/or therapeutic reviews, the following comparisons will be considered: no intervention, a placebo intervention, or an alternative intervention. Our primary adverse pregnancy outcomes of interests are maternal mortality, preterm delivery, and perinatal mortality. Two reviewers will independently identify eligible published and unpublished systematic reviews from six electronic databases and using hand searching of reference lists and citations. Data items extracted from included systematic reviews are based on the Cochrane Effective Practice and Organization of Care checklist and the preferred reporting items for systematic review and meta-analysis (PRISMA) statement. In our narrative data synthesis, we will consider risk of bias of individual reviews, focusing mainly on the conclusions of the highest quality reviews using the assessment of multiple systematic reviews (AMSTAR) checklist. Disagreements during search, selection, data extraction, and risk of bias assessment will be resolved through discussion and/or consultation of a third reviewer.

**Systematic review registration:**

PROSPERO CRD42015030132

**Electronic supplementary material:**

The online version of this article (doi:10.1186/s13643-016-0195-7) contains supplementary material, which is available to authorized users.

## Background

Periodontal disease is an inflammatory disease of the tissues supporting the teeth. In the USA, 65 million adults over the age of 30 years suffer from periodontal disease [1]. Five to seventy percent of adults worldwide have periodontal disease, depending on the geographical location and disease definition used [2]. Women who have periodontal disease while pregnant—an estimated one in five pregnant women—have been reported to be at increased risk of adverse pregnancy outcomes [3, 4].

The adverse pregnancy outcome most often associated with periodontal disease is preterm delivery [4]. A large prospective study by Offenbacher et al. showed that the prevalence of extremely preterm delivery (<28 weeks gestation) was increased tenfold in women with moderate-severe periodontal disease as compared to women without periodontal disease: 11.1 versus 1.1 %, respectively [5]. Other adverse pregnancy outcomes that have been associated with periodontal disease include low birth weight [5], preeclampsia [6, 7], and being small for gestational age (SGA) [8]. Although the association between periodontal disease and adverse pregnancy outcomes has been investigated in a considerable number of systematic reviews and meta-analyses, there are important differences in the conclusions of these reviews [9–12]. In line with this, systematic reviews assessing the effectiveness of therapeutic interventions to treat periodontal disease during pregnancy to try and reduce adverse pregnancy outcomes have arrived at different conclusions [13–15].

Given the global disease burden of both periodontal disease and the various adverse pregnancy outcomes that have been associated with it [16–18], a better understanding of the relationship between periodontal disease and adverse pregnancy outcomes, as well as the potential effectiveness of preventive and therapeutic interventions, is clearly needed in order to provide recommendations for clinical practice as well as guide future research into the possible underlying causal mechanisms.

## Objectives

We therefore aim to comprehensively assess the available evidence on this topic via a systematic overview of systematic reviews to address the following objectives:

- Summarize systematic reviews comparing the frequency of adverse pregnancy outcomes between women with and without periodontal disease
- Summarize systematic reviews evaluating the effect of preventive and therapeutic interventions for periodontal disease before or during pregnancy on adverse pregnancy outcomes.

## Methods/design

The research methods follow the preferred reporting items for systematic review and meta-analysis protocols (PRISMA-P; see also PRISMA-P checklist: Additional file 1) [19, 20]. Our study is registered with the PROSPERO prospective register of systematic reviews (CRD42015030132).

## Eligibility criteria

Studies will be selected according to the criteria outlined below.

## Study characteristics

### Study design

We will include systematic reviews: literature reviews that use a systematic search strategy to find relevant research aimed at answering a clearly defined clinical question. We will not include non-systematic narrative reviews, individual studies, case reports, case series, editorials, or clinical guidelines publications. Included reviews must describe a systematic search strategy and include data from either of the following: randomized controlled trials (RCTs, including cluster RCTs), controlled (non-randomized) clinical trials (CCTs) or cluster trials, interrupted time series (ITS) studies, (un)controlled before-and-after (CBA) studies, prospective or retrospective cohort studies, cross-sectional studies, and case-control or nested case-control studies.

### Participants

We will include systematic reviews reporting on studies in preconceptional or pregnant women with periodontal disease identified before (<6 months) or during pregnancy. Periodontal disease includes gingivitis (gingival inflammation) and periodontitis (gingivitis with gingival recession accompanied by loss of connective tissue and alveolar bone) [21, 22] and is usually identified by at least one of the following clinical periodontal indexes: clinical attachment loss, clinical attachment level, probing depth, or bleeding on probing [23].

### Interventions

Systematic reviews covering any intervention(s) aimed at prevention and/or treatment of periodontal disease before (<6 months) or during pregnancy are eligible for inclusion. Eligible interventions include (combinations of) the following: oral hygiene education, use of antibiotics, subgingival scaling, and root planing. Considering that we are also interested in the association between periodontal disease and adverse pregnancy outcomes irrespective of treatment, systematic reviews not covering any intervention may also be eligible for inclusion provided they report on this association.

### Comparisons

To evaluate the risk of adverse pregnancy outcomes in women with periodontal disease, we will include systematic reviews comparing the risk of adverse pregnancy outcomes between women with periodontal disease and women without periodontal disease before (<6 months) or during pregnancy. To evaluate the effect of preventive and/or therapeutic interventions for periodontal disease on adverse pregnancy outcomes, we will include systematic reviews comparing the risk of adverse pregnancy outcomes among women with periodontal disease during this period according to whether or not they received an intervention. The following will be considered as comparisons: no intervention, a placebo intervention, or an alternative intervention.

### Outcomes and prioritization

For the purpose of this overview, we define adverse pregnancy outcomes as a complication of pregnancy, labor, and delivery or post-partum period (up to 6 weeks after delivery) that is associated with maternal, fetal, and/or neonatal morbidity and/or mortality. Our primary and secondary outcomes of interest are listed below.

#### Primary outcomes

1. Maternal mortality (death during pregnancy or within 42 days after termination of pregnancy)
2. Preterm delivery (delivery of a live-born baby before 37 completed weeks of gestation)
3. Perinatal mortality (stillbirth or neonatal mortality (i.e., within 28 days after birth))

#### Secondary outcomes

1. Miscarriage (spontaneous abortion): fetal death before 20 weeks gestational age
2. Preterm prelabor rupture of membranes (PPROM): leakage of amniotic fluid in the absence of uterine activity before 37 weeks gestation
3. Pregnancy-induced hypertension: onset of hypertension (blood pressure ≥140/90 mmHg) in the second half of pregnancy in the absence of proteinuria or other markers of preeclampsia
4. Preeclampsia: hypertension and proteinuria
5. Clinical chorioamnionitis: clinical evidence of intra-amniotic infection with or without laboratory signs of infection
6. Histological chorioamnionitis: diagnosed by histological examination of the placenta by a pathologist [24]
7. Stillbirth (intrauterine death after 20 weeks of gestation)
8. Very preterm delivery (delivery of a live-born baby before 32 completed weeks gestation)
9. Low birth weight: birth weight of <2500 grams [25]
10. SGA: birth weight <10th centile for gestational age
11. Early onset neonatal sepsis: clinical evidence of sepsis with laboratory signs of infection within 72 h after delivery
12. Neonatal death: death of a baby occurring within the first 28 days of life [26]

We will consider other adverse pregnancy outcomes or composite outcomes for inclusion as relevant, emphasizing that these were not pre-defined.

## Information sources

Two reviewers will independently search for eligible systematic reviews through the following electronic databases (from inception): Cochrane Database of Systematic Reviews (CDSR), MEDLINE (Pubmed), EMBASE, World Health Organization (WHO), Global Health Library (covers African Index Medicus (AIM), Latin American and Caribbean Health Science Literature (LILACS), Index Medicus for the Eastern Mediterranean Region (IMEMR), Index Medicus for South-East Asia Region (IMSEAR), Western Pacific Region Index Medicus (WPRIM), and SciELO in addition to MEDLINE), and Google Scholar. This search will be supplemented by a search for unpublished, ongoing, or recently completed systematic reviews in PROSPERO. In addition, reference lists of included reviews will be screened to identify additional eligible systematic reviews. We will check for additional relevant articles by searching citations of eligible reviews via Google Scholar.

## Search strategy

A draft search strategy was developed for MEDLINE using medical subject headings (MeSH) and free text keywords related to pregnancy, periodontal disease, and systematic reviews (Additional file 2). The search strategy will be adapted for use in other databases. We will apply no restrictions to publication date or publication status of the review. Reviews published in any language will be eligible for inclusion.

## Study records

### Data management

All records identified will be uploaded or manually entered into EndNote X4.0.2 reference management software (Thomson Reuters, New York City, NY). We will automatically and manually de-duplicate records. EndNote will also be used during the selection process to keep track of the reasons for exclusion at different stages for presentation in the preferred reporting items for systematic review and meta-analysis (PRISMA) diagram.

## Selection process

Two reviewers will independently screen titles and abstracts of systematic reviews to assess eligibility for inclusion in this overview. After initial selection, full-text reports of potentially eligible systematic reviews will be obtained and screened to assess eligibility for final inclusion in the overview. In case of identification of unpublished or ongoing studies, we will contact the corresponding author for information on the current status of the systematic review (ongoing versus completed) and whether any preliminary data may be included in our overview. Disagreements will be resolved through discussion and/or consultation of a third reviewer. Reviewers will not be blinded to journal titles, study authors, or institutions.

## Data collection process

Two reviewers will independently extract relevant data from the included systematic reviews using a customized data extraction form. The data extraction form will be developed a priori and will be piloted using the first five eligible reviews and amended where required based on this pilot.

We will limit data extraction to the systematic reviews included in this overview and not pursue in-depth evaluation of the primary studies included in each systematic review. If relevant information is missing, we will contact authors of the included systematic reviews to request missing information.

The data items we will extract from each systematic review are informed by the Cochrane Effective Practice and Organization of Care checklist and the PRISMA statement [27, 28]. We will extract bibliographic and administrative data for each review, including author names and institutions, journal, publication year, funding sources and role of study sponsors, any conflicts of interest reported, and registration details of the review protocol, if applicable. We will extract the following data relating to the report characteristics of each review: eligibility criteria used, information sources used, the applied search strategy including search periods, applied limitations, and study selection methods. We will note the primary research questions and all outcomes for which data was sought, including length of follow-up where applicable. We will also extract the following main results of the review: the number of primary studies included, study population characteristics, and type of controls. In addition, for included systematic reviews that evaluated an intervention, we will extract details on the intervention(s) investigated: type of intervention, duration, frequency, timing, deliverer, setting, and for pharmacological interventions: generic and trade name and dosage. We will note any reported adverse effects of treatment. In case of meta-analysis having been conducted, we will extract relevant methodological aspects, as well as results of any meta-analyses, including subgroup and sensitivity analyses. We will also extract methodological details and results of any meta-bias assessments (e.g., assessment of publication bias) having been conducted. Finally, we will extract reported limitations and overall conclusions of the review. Disagreements during the data extraction process will be resolved through discussion and/or consultation of a third reviewer.

## Risk of bias for systematic reviews

There is currently no gold standard for the assessment of quality or risk of bias in overviews of systematic reviews [29, 30]. The most commonly used tool to assess the quality of systematic reviews included in overviews of systematic reviews is the assessment of multiple systematic reviews (AMSTAR) checklist [29]. Two reviewers will independently appraise risk of bias of the included systematic reviews using the validated AMSTAR checklist [31, 32] by scoring each systematic review with a maximum of 11 points. Disagreements will be resolved through discussion and/or consultation of a third reviewer. We plan to retain studies of any level of risk of bias in our data synthesis. We will, however, in our narrative synthesis consider risk of bias of individual reviews when discussing our findings, focusing mainly on the conclusions of the highest quality reviews.

## Data synthesis

In a narrative synthesis, we will summarize the characteristics and findings of the included systematic reviews using texts and tables to provide insight into the evidence for an association between periodontal disease and the evaluated outcome and, secondly, on how this association might be affected by periodontal treatment during pregnancy. The data extracted from the systematic reviews will be summarized and categorized according to pregnancy outcome. We will report any outcomes of interest for which no reviews were found.

We will not conduct a meta-analysis of meta-analyses. Pooling the results of the included systematic reviews is likely to introduce undesired bias as it will give disproportionate statistical power to primary studies included in more than one systematic review [33, 34].

## Risk of bias across systematic reviews

To provide insight in the degree of overlap in the inclusion of primary studies between systematic reviews, we will generate a citation matrix that cross-links all systematic reviews (columns) with all the primary studies included in the systematic reviews (rows) [34]. We will quantify the degree of overlap (i.e., the repeated occurrences of the primary studies) by calculating the corrected covered area (CCA) in this citation matrix [34].

To minimize publication bias, we aim to include all relevant data from unpublished ongoing or completed systematic reviews, as previously mentioned. To evaluate selective reporting within systematic reviews, we will compare outcomes reported in the systematic review protocols (if available) and the published reports of the systematic reviews. If a systematic review protocol is not available, we will compare outcomes reported in the methods and results sections of the published report of the systematic review.

## Confidence in cumulative estimate

We will explore the underlying reasons for any discrepancies between studies, taking into account study design characteristics and risk of bias. We will take these aspects into account when discussing the findings of our overview and formulating our conclusions.

## Discussion

This review will provide a synthesis of the existing systematic review literature on the association between periodontal disease and adverse pregnancy outcomes, as well as on the impact of interventions to prevent or treat periodontal disease on these outcomes. To the best of our knowledge, this will be the most comprehensive assessment of the evidence in this field to date. We will conduct and report our review using existing guidelines. A limitation of this undertaking is that the design (i.e., a review of reviews) precludes formulation of overall effect estimates via meta-analysis. Based on our evidence synthesis, we will formulate recommendations for clinicians caring for preconceptional and pregnant women. In doing so, our review has the potential to contribute to reducing the significant global burden of adverse pregnancy outcomes. We will furthermore identify the key knowledge gaps in the field and accordingly propose future research priorities.

## Ethics and dissemination

No formal ethical assessment or informed consent is required for the purpose of this study. In accordance with the PRISMA-P recommendations [19, 20], our protocol was registered with PROSPERO (http://www.crd.york.ac.uk/prospero/display_record.asp?ID=CRD42015030132) on 9 December 2015 (CRD42015030132). The findings of the study will be summarized in one or two manuscripts (i.e., one per research objective) to be submitted for publication in an international peer-reviewed scientific journal. In the event of amendments, reasons for deviation from the protocol will be mentioned in the definitive manuscript and will include a description of the changes made accompanied by the date of each amendment. Changes will not be incorporated into the protocol.

## Additional files

Additional file 1:
**The PRISMA-P checklist used while drafting the protocol manuscript.** (PDF 25 kb) Additional file 2:
**The search drafted and piloted for MEDLINE (Pubmed).** (PDF 14 kb)

## Abbreviations

AIM: African Index Medicus
AMSTAR: assessment of multiple systematic reviews
CBA: controlled before-and-after
CCA: corrected covered area
CCT: clinical controlled trial
CDSR: Cochrane Database of Systematic Reviews
IMEMR: Index Medicus for the Eastern Mediterranean Region
IMSEAR: Index Medicus for South-East Asia Region
ITS: interrupted time series
LILACS: Latin American and Caribbean Health Science Literature
MeSH: medical subject headings
PPROM: preterm prelabor rupture of membranes
RCT: randomized controlled trial
SGA: small for gestational age
WHO: World Health Organization
WPRIM: Western Pacific Region Index Medicus
