# Supplementary material for: Risk of adverse pregnancy outcomes in women with periodontal disease and the effectiveness of interventions in decreasing this risk: protocol for systematic overview of systematic reviews
Source: Syst Rev. 2016 Feb 1;5:16. doi: 10.1186/s13643-016-0195-7 (PMC4735974; doi:10.1186/s13643-016-0195-7)
Supplement: Additional file 2: — The search drafted and piloted for MEDLINE (Pubmed). (PDF 14 kb) [file 13643_2016_195_MOESM2_ESM.pdf]

Additional file 2: search strategy MEDLINE (Pubmed)

1. exp pregnancy/
2. pregnan*.mp
3. gestation*.mp
4. gravid*.mp
5. preconception*
6. conception
7. or/1-6
8. periodontal diseases/
9. gingival diseases/
10. exp gingivitis/
11. exp periodontitis/
12. periodontal index/
13. oral health/
14. periodontal disease*.mp
15. gingiv*.mp
16. gum disease*.mp
17. “gingival health”.mp
18. swollen gum*.mp
19. “bleeding on probing”.mp
20. periodont*.mp
21. parodont*.mp
22. paradont*.mp
23. “periodontal index”.mp
24. “oral health”.mp
25. “oral disease”.mp
26. or/8-25
27. review/
28. meta-analysis/
29. “systematic review”.mp
30. review.mp
31. meta-analy*.mp
32. metaanaly*.mp
33. or/27-32
34. 7 AND 26 AND 33
